# RPA-assisted CRISPR-Cas12a-enabled point-of-care diagnostic platform for chili leaf curl virus with fluorescent and colorimetric readouts

**DOI:** 10.3389/fmicb.2025.1644322

**Published:** 2025-10-15

**Authors:** Samrat Paul, Venu Emmadi, Shipra Saxena, Mehulee Sarkar, Bikash Mandal, Ravinder Kumar, Parimal Sinha, Anirban Roy

**Affiliations:** Advanced Centre for Plant Virology, Division of Plant Pathology, ICAR-Indian Agricultural Research Institute, New Delhi, India

**Keywords:** chili leaf curl virus, on-site detection, CRISPR-Cas12a, DETECTR, recombinase polymerase amplification, lateral flow assay

## Abstract

Chili leaf curl virus (ChiLCV) is a highly destructive begomovirus that causes significant economic losses in chili production across the Indian subcontinent. Accurate detection of the virus is crucial for effective disease management. This study presents a Recombinase Polymerase Amplification (RPA)-assisted DNA endonuclease-targeted CRISPR trans reporter (DETECTR) system for the rapid, highly sensitive, and specific detection of ChiLCV, specifically targeting the *AC1* gene sequence. A crRNA specific to *AC1* gene of ChiLCV- was designed, and the RPA conditions were optimized. The detection method involves cleaving a tagged oligo reporter (Fluorophore-quencher or Biotin-FAM), allowing results to be visualized via either a fluorescence read-out-based assay or a Lateral Flow Assay (LFA) with gold nanoparticles conjugated to FAM antibody. We standardized the critical concentration of the biotin-FAM oligo reporter such that, in the presence of the viral genome, the activated CRISPR-Cas12a cleaves all reporters, resulting in a dark test line on the lateral flow strip. This RPA-assisted fluorescence or LFA readout-based DETECTR system demonstrates exceptional specificity and sensitivity, detecting ChiLCV at a concentration as low as 7 femtograms when using cloned plasmid DNA, comparable to the gold standard detection method, like real-time PCR. The system successfully detected the virus in crude leaf extracts from infected plants while distinguishing ChiLCV from related begomoviruses and damage caused by common pests like mites and thrips. The DETECTR system was finally validated with field infected samples collected from major chili-growing states of India. To the best of our knowledge, this is the first demonstration of a CRISPR-based assay for ChiLCV that can be applied directly to crude leaf extracts, thereby enhancing its potential utility in point-of-care diagnostics. A key advantage of this diagnostic approach is its rapid processing time and field applicability, making it an accessible and practical tool for farmers and agricultural specialists to implement timely virus disease management strategies for chili crops.

## 1 Introduction

Chili peppers (*Capsicum annuum*) represent a crucial crop in India, possessing significant economic and cultural importance. With an annual production of 2.7 million tons and a productivity rate of approximately 8.58 kg/ha (Food and Agriculture Organization of the United Nations; 2023^1^, verified on 03.09.2025), India stands as the largest producer and exporter of this crop, contributing 25% of the global production. However, in recent years, shifts in climatic conditions and the resurgence of pests have resulted in the spread of whitefly-transmitted begomoviruses, which cause leaf curl disease and lead to significant production losses. Symptoms, including upward curling, puckering, and a reduction in leaf size, characterize the disease. Severely affected plants may appear stunted and may not bear fruit (Senanayake et al., 2007). When infection occurs early during crop growth, it can lead to yield reductions ranging from 85 to 100%, resulting in a significant economic impact that threatens farmers’ livelihoods and destabilizes the agricultural economy (Kumar et al., 2011; Senanayake et al., 2012; Thakur et al., 2018). In the Indian subcontinent, the disease is caused by various whitefly-transmitted begomoviruses. Some of these have a bipartite genome (DNA-A and DNA-B), while others possess a monopartite circular single-stranded DNA that is closely related to DNA-A of bipartite begomoviruses. Often, such monopartite begomoviruses are also associated with a betasatellite (Chattopadhyay et al., 2008; Kumar et al., 2015; Kushwaha et al., 2015). Among these begomoviruses, the species *Begomovirus chillicapsici*, commonly known as the chili leaf curl virus (ChiLCV), poses a significant threat to the cultivation of this crop (Roy et al., 2021). In addition to ChiLCV, chili crops are also susceptible to damage from thrips and mites. The excessive feeding by these pests often leads to leaf smalling and deformities, which can easily be mistaken for symptoms of leaf curl (Bala et al., 2015; Sharma et al., 2009). This misdiagnosis not only results in the implementation of ineffective management practices but also places a financial burden on farmers’ cultivation costs.

Traditional diagnostic approaches for ChiLCV include PCR (Jayanthi et al., 2024), real-time PCR (qPCR) (Kumar et al., 2022), loop-mediated isothermal amplification (LAMP) (Catherine et al., 2024; Ashwathappa et al., 2025), and localized surface plasmon resonance (LSPR) assays (Das et al., 2021). Among these, qPCR remains the gold-standard laboratory method, with a detection limit of ∼0.15 fg of viral DNA (∼1.7 × 10^−16^ M) from extracted DNA (Kumar et al., 2022). While these methods provide reliable and sensitive detection, they generally require specialized equipment, skilled personnel, and relatively long assay times, which limit their applicability for on-site or field-level diagnostics. Therefore, there is an urgent need for rapid and accessible diagnostic tools that can be easily employed in the field (Boluk et al., 2020; Dyussembayev et al., 2021). Recently, innovative diagnostic methods based on clustered regularly interspaced short palindromic repeats (CRISPR) and CRISPR-associated proteins (Cas) systems have emerged as promising solutions for rapid and accurate detection. One notable system is the DNA-endonuclease-targeted CRISPR trans reporter (DETECTR), which has been effectively utilized for Cas12a-assisted nucleic acid detection (Chen et al., 2018; Gootenberg et al., 2018). Cas12a is an RNA-guided nuclease that cleaves double-stranded DNA containing a thymine-rich protospacer adjacent motif (PAM). Following this cleavage, Cas12a non-specifically cleaves surrounding single-stranded DNA (ssDNA), referred to as reporter DNA (Zetsche et al., 2015; Bernabé-Orts et al., 2022).

The DETECTR system requires a critical concentration of template DNA, which typically necessitates a pre-amplification step to achieve this concentration. This pre-amplification of template DNA can be accomplished using either loop-mediated isothermal amplification (LAMP) or recombinase polymerase amplification (RPA) techniques. When combined with these pre-amplification methods, DETECTR has been successfully employed for the highly sensitive and specific detection of various plant viruses, including potato virus X (PVX), potato virus Y (PVY), tobacco mosaic virus (TMV), as well as multiple RNA viruses and viroids infecting apple, tomato yellow leaf curl virus (TYLCV), tomato leaf curl New Delhi virus (ToLCNDV), beet necrotic yellow vein virus (BNYVV), and tomato brown rugose fruit virus (ToBRFV) (Aman et al., 2020; Jiao et al., 2021; Mahas et al., 2021; Bernabé-Orts et al., 2022).

This study presents the development of an RPA-assisted CRISPR-Cas12a-based DETECTR system designed for fluorescence readout or lateral flow assays, enabling rapid, highly sensitive, and specific detection of ChiLCV.

## 2 Materials and methods

### 2.1 Virus construct, plant genotypes and method of virus inoculation

A partial tandem repeat-based agroconstruct (PTR) derived from the ChiLCV Maharashtra isolate (MK882926) was employed in this study to standardize diagnostic methods, as described previously (Shilpi et al., 2015; Roy et al., 2019). For agroinoculation, the PTR construct of ChiLCV was mobilized into the Agrobacterium strain EHA105 and was grown in Luria agar for 48 h at 28 °C and then harvested in 500 μl of B5 medium. The Agrobacterium culture was prick-inoculated to young seedlings (3–4 leaf stage) of Pusa Jwala cultivar of chili, recognized for its susceptibility to ChiLCV, and grown under controlled environmental conditions (24 ± 2 °C and 70% relative humidity). In the study, cloned DNA of ChiLCV was used primarily for assay standardization, as well as for specificity and sensitivity assessments. Symptomatic leaf samples from agroinoculated plants served as the test material. However, for field validation, both symptomatic and asymptomatic chili leaf samples, as well as those suspected to be symptomatic due to virus, mite, or thrips damage, were collected from major chili-growing regions across India, including experimental research fields in New Delhi and farmers’ fields in Telangana, Andhra Pradesh, Karnataka, and West Bengal. This approach ensured a comprehensive representation of the virus strains. Ten samples were collected from each location.

### 2.2 CRISPR RNA (crRNA) designing

ChiLCV and other begomovirus sequences were retrieved from NCBI^2^. To ensure inclusivity across diverse isolates of ChiLCV existing in India, the crRNA target site was carefully selected from a highly conserved region of the *AC1* gene of ChiLCV, which is reported to be conserved among all Indian isolates. This design consideration provides confidence that the developed assay is broadly applicable to naturally occurring ChiLCV variants while minimizing the risk of false negatives. To pinpoint highly conserved regions within the *AC1* gene, the *AC1* gene sequences of all ChiLCV isolates were aligned using BioEdit software (version 7.2) (Hall, 1999). Putative crRNA sequences were identified from such conserved stretches of *AC1* gene using EuPaGDT web tool^3^ and were further analyzed to evaluate their suitability based on off-target with chili genome, free energy and GC content, which are crucial factors for stable crRNA-DNA interaction during the CRISPR-Cas12a detection. Secondary structure of the potential crRNAs was obtained through RNAfold server^4^. Ultimately, the most suitable crRNA sequence was aligned with other closely related begomovirus genomic sequences to confirm its specificity for ChiLCV. The final crRNA sequences, consisting of 21 nucleotides (excluding the PAM sequence), were synthesized along with a conserved loop sequence (20 nucleotides) necessary for recognition by LbaCas12a, utilizing the commercial services of Integrated DNA Technologies, Inc., USA.

### 2.3 Evaluation of crRNA

To evaluate the efficiency of the crRNA, an *in vitro* cleavage assay was performed. First, the genome of ChiLCV was rescued by restriction digestion of the partial tandem repeat-based agroconstruct of the ChiLCV Maharashtra isolate. The retrieved ChiLCV genomic DNA (target DNA) was then incubated at 37 °C for 30 min with the synthesized crRNA sequence and EnGen^®^ Lba Cas12a (New England Biolabs, USA) in the molar ratio of 1:10:10 in the presence of 1X R2.1 buffer. The resultant product was evaluated in 1.5% agarose gel electrophoresis. To visualize the efficacy of crRNA for the detection of the ChiLCV genome, 50 nm of DNase alert (hex reporter) (New England Biolabs, USA) was added to the crRNA-Cas12a-template reaction mix (1:10:10) and incubated at 37 °C for 30 min. Fluorescence was observed under UV light in a gel documentation system. To test the specificity of the synthesized crRNA, a similar reaction was performed with cloned genomic DNA of six other closely related begomoviruses, *viz.* tomato leaf curl New Delhi virus (ToLCNDV), tomato leaf curl Palampur virus (ToLPalV), tomato leaf curl Joydebpur virus (TolCJoV), tomato leaf curl Gujarat virus (ToLCGuV), tomato leaf curl Bangalore virus (ToLCBV), and tomato leaf curl Karnataka virus (ToLCKV). The sensitivity of CRISPR-Cas12a for detecting ChiLCV was evaluated using a cloned viral genome, total DNA, and a crude leaf extract isolated from ChiLCV infected plants. ChiLCV-infected plant DNA was obtained from the symptomatic leaves of ChiLCV agroinoculated plants and extracted using the CTAB method as standardized in our laboratory (Roy et al., 2019). A crude leaf extract from these symptomatic leaves was prepared in 1X TE (pH 8.0) buffer. The CRISPR-Cas12a-DNase alert complex was incubated with 100 ng (1 μl) of total DNA and 1 μl of the crude leaf extract, and fluorescence was recorded as previously described. In all experiments, the cloned ChiLCV genome and a reagent control (without DNA) served as positive and negative controls, respectively.

### 2.4 Evaluation of RPA

A pair of RPA primers (FP2: 5′ggaagatagcgggaattccaccttt aatttga3′ and RP7: 5′tctgccaacgacgcatatgccgaggcaatca3′) were designed from the *AC1* gene of the ChiLCV Maharashtra isolate to amplify a ca. 520 bp amplicon containing the crRNA sequence. The RPA reactions were performed using the TwistAmp Basic Kit (TwistDX, UK) according to the manufacturer’s protocol. Specifically, for each 50 μl of RPA mastermix, 29.5 μl of Rehydration Buffer (10X), 2.4 μl of forward primer (10 μM), 2.4 μl of reverse primer (10 μM), and 12.5 μl of nuclease-free water were combined with 1 μl of the template (ChiLCV-cloned DNA) and 2.5 μl of 280 mM MgOAc. RPA reactions were standardized across various temperatures (32° C, 35° C, 37° C, 39° C, and room temperature) and durations (5, 10, 15, 20, and 25 min) using a dry bath incubator (Helix Biosciences, India), to determine the optimal conditions for enzyme activity and DNA amplification. To assess the detection limit of the RPA, ChiLCV-cloned DNA was serially diluted from 0.3 ng to 30 attograms, and the RPA reaction was conducted as previously described. To evaluate the sensitivity of the RPA with total DNA and crude sap, total infected plant DNA was serially diluted from 100 ng to 1 femtogram, and crude leaf extract was diluted 10-fold up to 10^–5^ dilutions. The sensitivity of RPA was compared with that of PCR using identical DNA dilutions. For PCR, the same primers as in RPA were used to amplify the ChiLCV genomic fragment, with an annealing temperature of 58 °C and 35 amplification cycles. Additionally, the specificity of the RPA reaction was tested using cloned DNA from six other closely related begomoviruses, as previously mentioned. The resulting RPA products were resolved on a 1.5% agarose gel.

### 2.5 RPA-assisted CRISPR-Cas12a-fluorescence assay

For RPA-assisted CRISPR-Cas12a-fluorescence assay, RPA reaction was carried out from the total DNA as well as crude sap as described earlier. A volume of 1 μl from the RPA amplified product was then incubated with CRISPR-Cas12a-DNase alert at 37 °C for 15–20 min as previously described. Total DNA and crude extract from three individual samples were tested. Cloned ChiLCV genome served as the positive control, while total DNA from a healthy plant was used as the negative control.

### 2.6 RPA-assisted CRISPR/Cas12a-lateral flow assay

The lateral flow assay (LFA) utilized the HybriDetect-1 kit (Milenia Biotec) and a 5′-FAM-TTATT-3′-Biotin reporter. The LFA strips feature two distinct lines: a control line (positioned first in the direction of flow) composed of Streptavidin that can bind to its affinity partner Biotin of the FAM-Biotin reporter, and a test line comprising anti-rabbit IgG raised against the FAM antibody. The sample pad contains Au-nanoparticle conjugated anti-FAM antibodies that flow along the nitrocellulose membrane via capillary action. The control line captures the intact FAM-Biotin reporter molecules through affinity binding until they are cleaved by the crRNA-cas12a complex.

To determine the optimal reporter concentration, a 100 μM stock of FAM-Biotin reporter was diluted with nuclease-free water across two concentration ranges: a lower range comprising 5 nM, 500 pM, 50 pM, and 5 pM; and a higher range comprising 100 nM, 200 nM, 300 nM, 400 nM, and 500 nM. Subsequently, 1 μl of the RPA-amplified product was incubated with CRISPR-Cas12a-FAM-Biotin-oligo reporter at 37° C for 15–20 min as previously described. Following incubation, 80 μl of dipstick assay buffer was added to 20 μl of the mixture before application to the sample pad. Results were typically visible within 5 min.

To evaluate the sensitivity of the RPA-assisted CRISPR-Cas12a-based LFA, ChiLCV-cloned DNA was serially diluted from 70 ng to 7 fg and tested. Furthermore, the specificity of the on-spot detection system was assessed using cloned DNA from six other closely related begomoviruses, as mentioned earlier. The relative intensity of the band at the test line (T) compared to the control line (C), expressed as the T/C ratio, was calculated using ImageJ software.

### 2.7 Logistic threshold modeling for reporter optimization

The appearance of bands on the LFA strip relies entirely on the binding of Au-nanoparticle conjugated anti-FAM antibodies to the respective lines on the strip. Specifically, binding at the control line (C) depends on the presence of the FAM-Biotin oligo reporters in the reaction mixture. Estimation of the proper concentration of reporters is essential for visualizing distinguishable bands under both positive and negative conditions. Notably, because a single activated CRISPR complex can cleave multiple reporter molecules, the visibility of the system is not primarily governed by the amount of CRISPR complex or target DNA but rather by how many intact reporters remain available to interact with the detection lines. Based on relative intensity (T/C) values at different reporter concentrations as mentioned earlier, Logistic Threshold Modeling was performed for selection of optimal concentration for FAM-Biotin reporter. A four-parameter logistic function was applied separately to the positive and negative datasets to model the nonlinear response of the system (DeLean et al., 1978; Motulsky and Christopoulos, 2004):


Response=

$$\;B\,a\,s\,e\,l\,i\,n\,e\,r\,e\,s\,p\,o\,n\,s\,e+\frac{\left(M\,a\,x\,i\,m\,u\,m\,r\,e\,s\,p\,o\,n\,s\,e-B\,a\,s\,e\,l\,i\,n\,e\,r\,e\,s\,p\,o\,n\,s\,e\right)}{1+10^{\left(\text{log}\,E\,C\,50-\text{log}\,\left[R\right]\right).H\,i\,l\,l\,s\,l\,o\,p\,e}}$$

Where, *Baseline response* = minimum T/C value, *Maximum response* = maximum T/C value, *[R]* = reporter concentration (nM), *EC50* = concentration where T/C is halfway between min and max, *Hill slope* = Hill slope indicating a steep transition near the threshold.

### 2.8 Statistical analysis

Diagnostic performance was assessed using receiver operating characteristic (ROC) curve analysis. All statistical analyses were conducted using R Statistical Software (version 4.3.1) [29]. Multilocation data were validated using the Chi-square test, and bar plots were generated to visualize categorical variables. Statistical analyses were performed to ensure robust interpretation and reliability of the experimental findings. All experiments were carried out in triplicate to account for reproducibility and minimize variability.

### 2.9 Cost component analysis

To evaluate the practical applicability of the DETECTR assay, we conducted a comparative cost component analysis against conventional end-point PCR, SYBR Green-based qPCR, and probe-based qPCR. The following cost categories were included: nucleic acid preparation, amplification reagents, CRISPR/Cas components (Cas12a, crRNA, reporter), detection readout (gel electrophoresis, fluorescence, or lateral flow strips), primers/probes, consumable plastics, labor (estimated on a batch of ∼24 samples), overheads (15% of consumables), and equipment amortization. Costs were estimated using current commercial prices from standard suppliers in India (2024 rates) and converted into USD (based on Rs. 88.08/- for 1 USD as on 03.09.25). Equipment amortization was calculated assuming a 5-year lifespan with an average throughput of ∼3,000 samples annually. Final estimates are expressed as approximate cost per sample for cross-platform comparison.

## 3 Results

### 3.1 crRNA-Cas12a alone is effective in detecting ChiLCV from the cloned plasmid but not from DNA or crude extract

**FIGURE 1:**
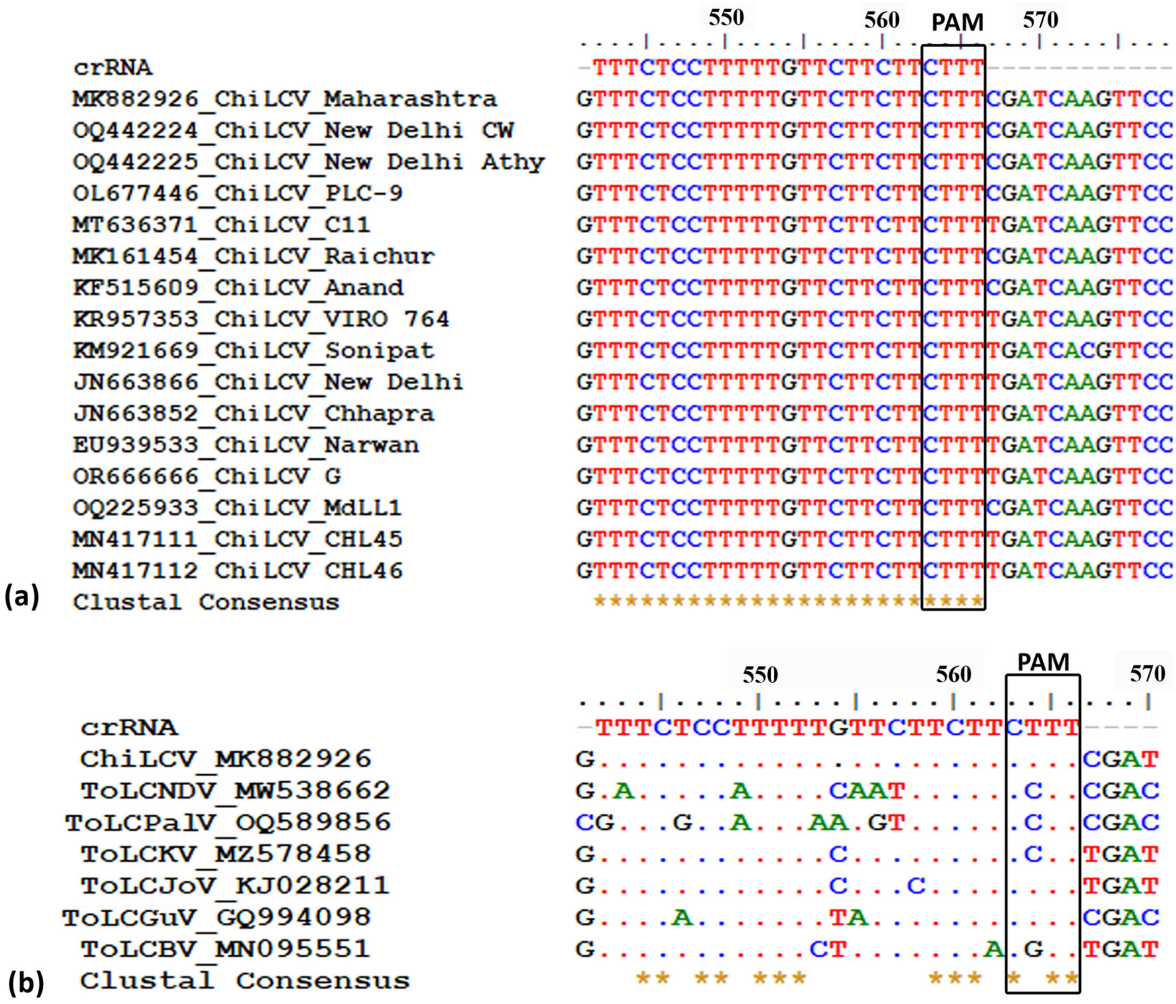
Designing potential crRNA targeting conserved *AC1* region of chili leaf curl virus (ChiLCV). **(a)** Alignment of *AC1* region of different chili leaf curl virus isolates to identify the conserved region. **(b)** Determination of off-target effect against tomato leaf curl new Delhi virus (ToLCNDV), tomato leaf curl Palampur virus (ToLPalV), tomato leaf curl Karnataka virus (ToLCKV), tomato leaf curl Joydebpur (ToLCJoV), tomato leaf curl Gujarat virus (ToLCGuV), and tomato leaf curl Bangalore virus (ToLCBV).

Through a comprehensive screening process, we evaluated nine potential crRNAs targeting a conserved region of the *AC1* gene in ChiLCV, meticulously assessing several critical parameters as outlined in Table 1. Among the nine crRNAs, only one sequence (5′ TTTCTCCTTTTTGTTCTTCTTCTTT 3′) demonstrated significant sequence conservation across the tested ChiLCV isolates (Figure 1a). This crRNA also exhibited a favorable secondary structure lack of any hairpin structure (Supplementary Figure 1, structure 9) and displayed sequence differences in the corresponding homologous regions of closely related begomoviruses, highlighting its specificity for ChiLCV (Figure 1b).

**TABLE 1:** Bioinformatic analysis of crRNA designed from *AC1* conserved region of ChiLCV.

Rank	Target sequence	Location in virus genome	Strand	GC content (%)	Total score	Off-target in chili genome	Off-target with other begomoviruses	Conserveness with other ChiLCV isolate	Potential problems during transcription	Secondary structure Free energy (kcal/mole)
1	TTTTTACGCCTCCG TTGGAGGTTTA	2072–2096	–	52	0.58	0	0	No	No problem found	−8.3
2	TTTCGATCAAGT TCCGGAGGAACTT	2023–2047	–	48	0.57	0	0	No	No problem found	−6.2
3	TTTACGCCTCCGTT GGAGGTTTATG	2070–2094	–	57	0.56	0	0	No	No problem found	−8.3
4	TTTGTTCTTCTTCT TTCGATCAAGT	2036–2060	–	33	0.54	0	0	No	gRNA does not start with “G” or “A”, please manually add a leading “G” or “A”	3.9
5	TTTTACGCCTCCGT TGGAGGTTTAT	2071–2095	–	52	0.53	0	0	No	gRNA does not start with “G” or “A”, please manually add a leading “G” or “A”	−8.3
6	TTTTTGTTCTTCT TCTTTCGATCAA	2038–2062	–	33	0.52	0	0	No	gRNA does not start with “G” or “A”, please manually add a leading “G” or “A”	0
7	TTTATGTTTCT CCTTTTTGTTCTTC	2051–2075	–	33	0.5	0	0	No	No problem found	0
8	TTTTGTTCTTCTT CTTTCGATCAAG	2037–2061	–	38	0.49	0	0	No	gRNA does not start with “G” or “A”, please manually add a leading “G” or “A”	2.7
9	TTTCTCCTTTTT GTTCTTCTTCTTT	2045–2069	–	29	0.48	0	0	Yes	No problem found	0

**FIGURE 2:**
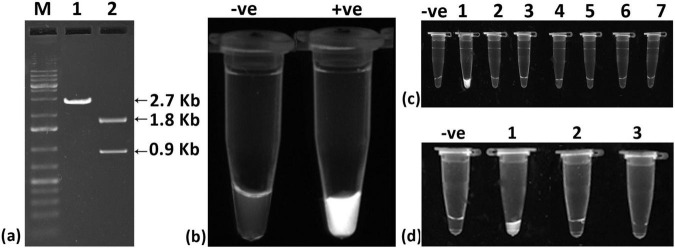
Evaluation of designed crRNA for detection of chili leaf curl virus (ChiLCV). **(a)** *In vitro* Cleavage assay of crRNA-Cas12a targeting ChiLCV genome; Lane 1: cloned plasmid of ChiLCV gave expected fragment size of 2.7 Kb, Lane2: cloned plasmid of ChiLCV plus crRNA-CAS12a complex gave two fragments of 1.8 Kb and 0.9 Kb, Lane M: Molecular Markar **(b)** Fluorescence-based detection of ChiLCV; Tube 1: Blank (no fluoresecence), Tube 2: cloned plasmid of ChiLCV plus crRNA-CAS12a complex (strong fluorescence signal). **(c)** Specificity assay showing the system is specific to ChiLCV detection; Blank: –ve control, Tube 1: cloned plasmid of ChiLCV plus crRNA-CAS12a complex, Tube 2: ToLCNDV genomic DNA plus crRNA-CAS12a complex, Tube 3: ToLPalV genomic DNA plus crRNA-CAS12a complex, Tube 4: ToLCJoV genomic DNA plus crRNA-CAS12a complex, Tube 5: ToLCGuV genomic DNA plus crRNA-CAS12a complex, Tube 6: ToLCBV genomic DNA plus crRNA-CAS12a complex, and Tube 7: ToLCKV genomic DNA plus crRNA-CAS12a complex. **(d)** crRNA-Cas12a based detection could not detect viral DNA from total DNA isolated from infected plant and crude sap; Blank: –ve control), Tube 1: ChiLCV cloned plasmid plus crRNA-CAS12a complex (+ve control), Tube 2: total DNA extract plus crRNA-CAS12a complex, Tube 3: crude extract from infected leaf plus crRNA-CAS12a complex.

The selected crRNA, in combination with Cas12a, successfully cleaved the cloned viral genome (∼2.7 kb) into two fragments of approximately 1.8 kb and 0.9 kb (Figure 2a), demonstrating the effectiveness of the crRNA-Cas12a system. Further assessment through fluorescence testing revealed fluorescence when the cloned viral genome was used (Figure 2b). In specificity tests, fluorescence was detected exclusively with the cloned ChiLCV viral genome, confirming that the crRNA is specific to ChiLCV (Figure 2c). To evaluate the sensitivity of the crRNA-Cas12a system independently, we tested it with total DNA and crude sap from infected chili plants. However, the system showed limited detection capabilities, responding only to the cloned viral genome, thereby indicating potential sensitivity limitations when solely relying on the crRNA-Cas12a system (Figure 2d).

### 3.2 RPA alone is highly sensitive but not specific for detecting ChiLCV

**FIGURE 3:**
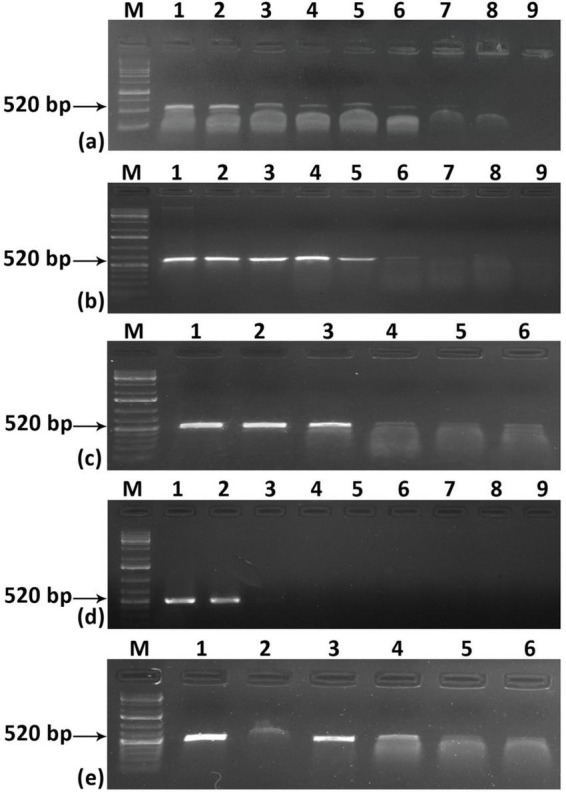
Sensitivity and specificity assay for RPA-based detection of chili leaf curl virus (ChiLCV). Sensitivity was assessed using different concentrations of **(a)** cloned viral plasmid **(b)** total DNA isolated from an infected plant, and **(c)** crude leaf extract. **(d)** comparison of the sensitivity of PCR. **(e)** test of specificity of RPA-based detection. Lane M: Molecular Marker.

To address the aforementioned limitation regarding the sensitivity of the system, we employed a Recombinase Polymerase Amplification (RPA)-based detection method. The RPA could able to detect the virus in a wide range of temperatures, ranging from 35–39° C and within 10 min when total DNA was used as a template (Supplementary Figures 2a, b). For crude extract also ChiLCV could be detected at a similar temperature range but it took a minimum of 10 min to amplify the viral fragment (Supplementary Figures 2 c, d). The RPA primers we designed demonstrated remarkable sensitivity across various sample types. With the cloned viral genome, the sensitivity was exceptionally high, detecting as little as 0.03 fg (approximately 1000 copies of the viral genome) (Figure 3a). In contrast, when using total DNA obtained from ChiLCV agroinoculated plants, the detection threshold was 10 pg (Figure 3b). Standard PCR-based testing, by comparison, was only able to detect ChiLCV from the same total DNA up to 10 ng (Figure 3d). Notably, the RPA method successfully detected ChiLCV even from a 10-3 dilution of crude leaf extract from infected plants (Figure 3c). However, during specificity testing with the cloned viral genomes of six closely related begomoviruses, RPA resulted in amplifications from multiple begomoviruses. This suggests a potential non-specificity issue of RPA for begomovirus detection due to the close similarity of the RPA primers with other tested begomoviruses (Figure 3e). This cross-reactivity highlights the necessity for further refinement to improve specificity.

### 3.3 RPA-assisted CRISPR Cas12a system improves the specificity and sensitivity

**FIGURE 4:**
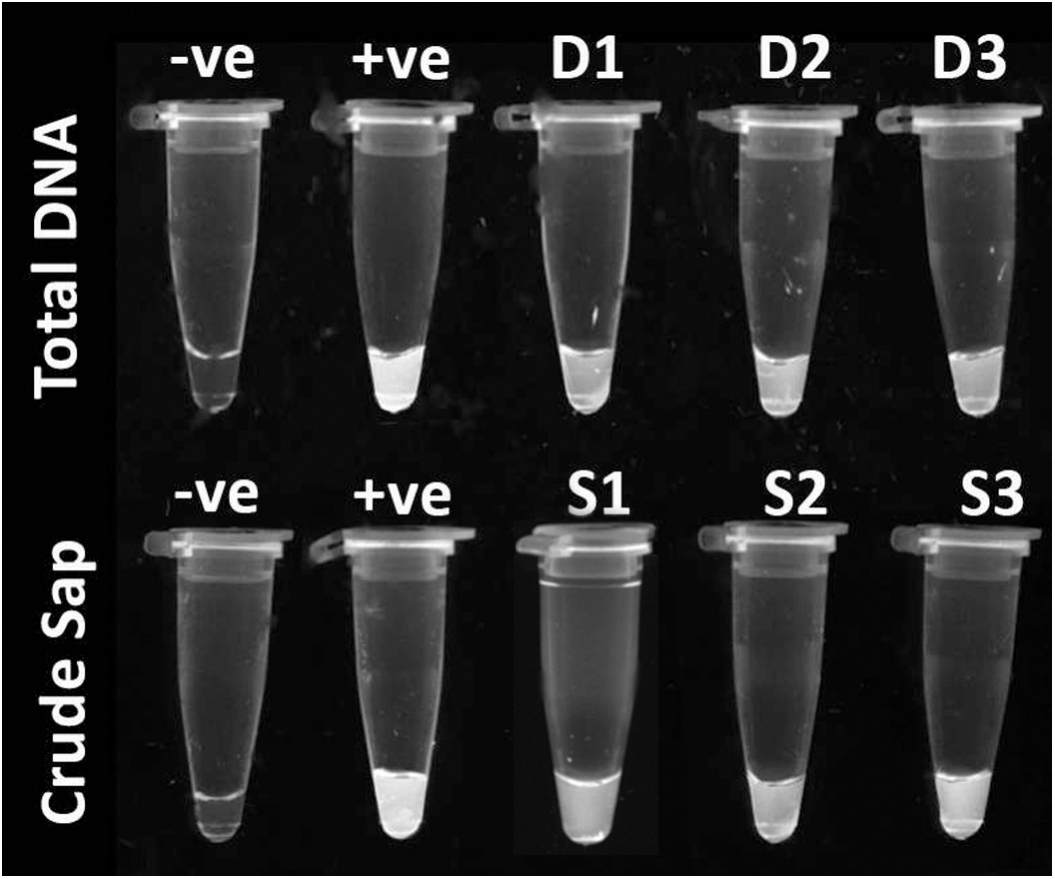
Recombinase Polymerase Amplification assisted CRISPR-Cas12a based detection of ChiLCV from total DNA (first row), –ve control: healthy sample, +ve: ChiLCV plasmid and D1, D2, D3: total DNA from infected plants and; from crude sap (second row), –ve control: healthy sample, +ve: ChiLCV plasmid, S1, S2, S3: crude extract from infected sample.

Integration of high sensitivity of Recombinase Polymerase Amplification (RPA) with increased specificity of the CRISPR system gave a versatile detection strategy. This hybrid system can detect targets in both total DNA from infected plants and crude sap with high specificity (Figure 4).

### 3.4 FAM-Biotin reporter concentration determines the visual output of detection of ChiLCV

**FIGURE 5:**
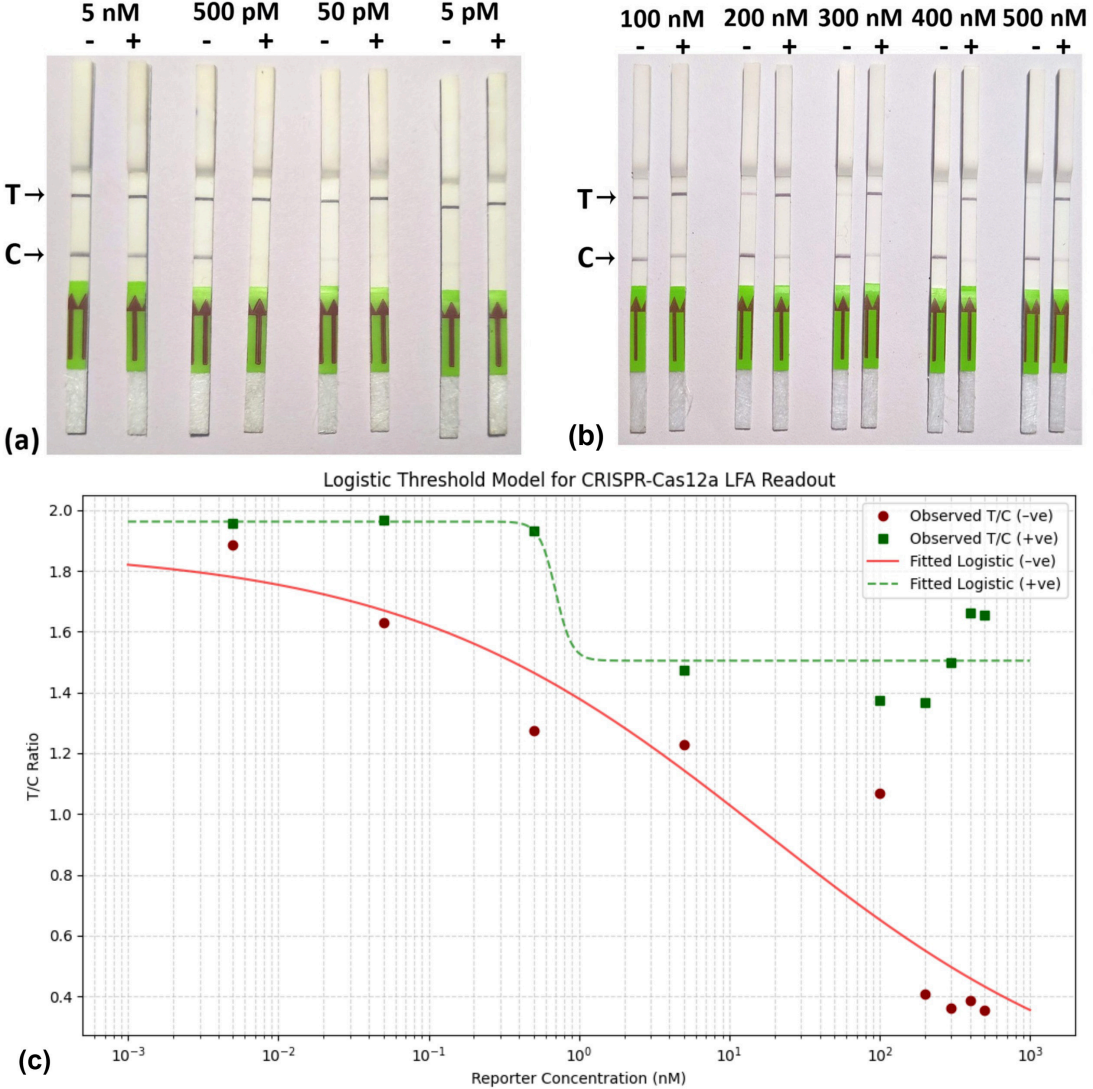
Standardization of RPA-CRISPR-cas12a based Lateral flow assay. **(a)** Reaction mixture, containing RPA product, CRISPR-cas12a mix, and 5 different low concentrations (5 nM, 500 pM, 50 pM, 5 pM) of FAM-Biotin reporter was incubated and applied to the lateral flow strip. **(b)** Reaction mixture, containing RPA product, CRISPR-cas12a mix, and 5 different high concentrations (100 nM, 200 nM, 300 nM,400 nM,500 nM) of FAM-Biotin reporter was incubated and applied to the lateral flow strip. **(c)** Logistic Threshold Model for Reporter concentration where X axis represents reporter concentration (nM); Y axis represtents relative intensity value of T/C.

At low concentrations of the reporter, ranging from 5 nM to 5 pM, a concentration of 500 pM was found to be effective for the threshold for visible change (Figure 5a), Conversely, at higher reporter concentrations (200 nM and above), the LFA demonstrated a clear capability to distinguish between the presence of the virus and healthy samples (Figure 5b). Furthermore, the observation is strengthening by the Logistic Threshold Modeling (Figure 5c), where the maximum visible difference of T/C values between the positive samples and control was observed at 500 nM. The model shows that below ∼300 nM, the T/C ratio for negatives can overlap with positives due to insufficient reporter binding, leading to false results, a T/C below 0.5 is deemed negative. The visual output of the Fam-Biotin reporter-based LFA assay is influenced by the concentrations of the reporter and the virus. In scenarios where the reporter concentration is low, three possibilities may arise: (i) the absence of the virus, (ii) the presence of the virus at a low concentration, and (iii) the presence of the virus at a high concentration.

**FIGURE 6:**
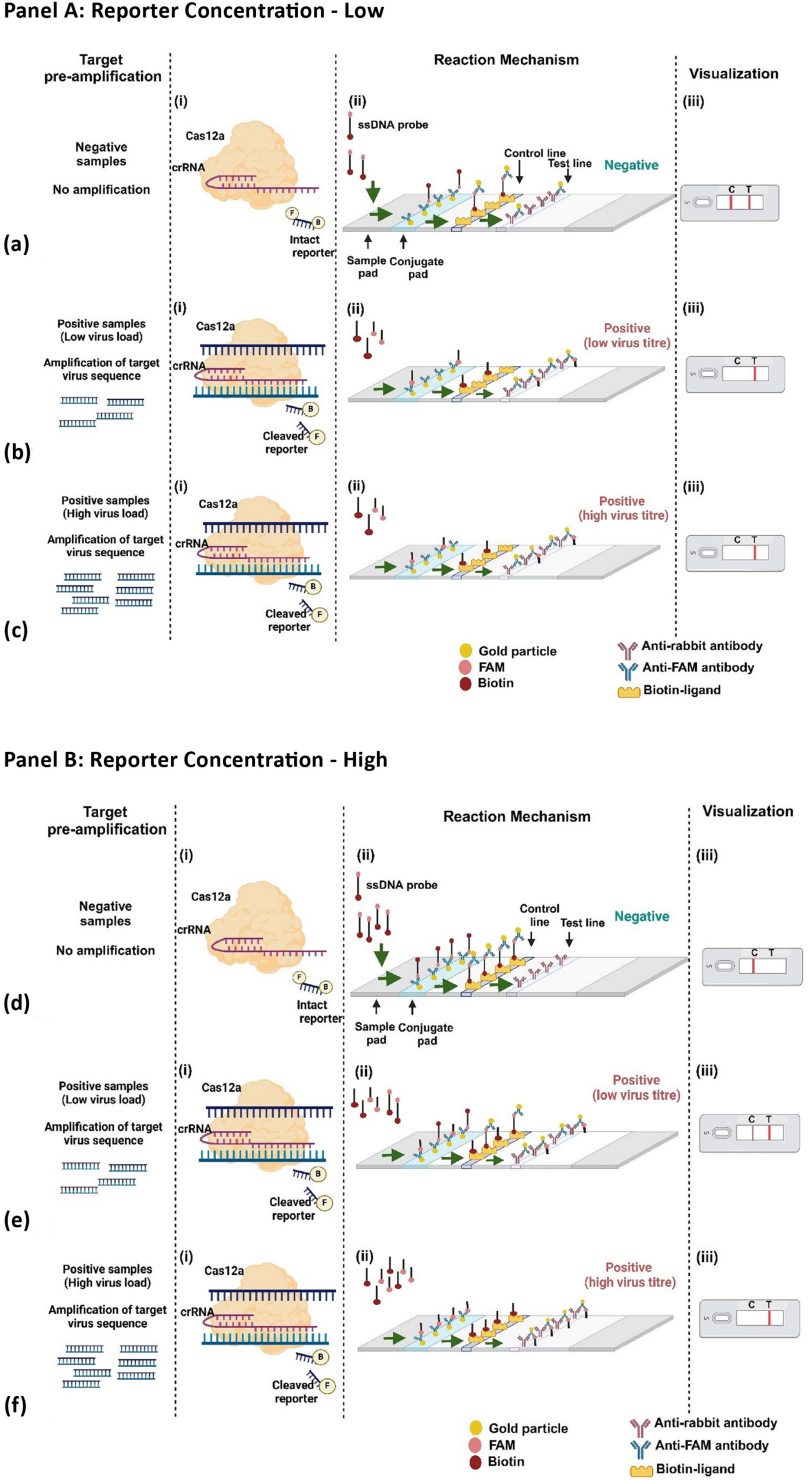
Workflow of CRISPR-Cas12a with FAM-Biotin reporter at varying reporter concentration (Panel [**A]:** low reporter concentration; Panel [**B]:** high reporter concentration).

In the first scenario, where the virus is absent, the CRISPR-Cas12a complex remains inactive, resulting in no cleavage of the FAM-Biotin reporter (Figure 6A, a). This leads to the formation of two bands at both the C- and T-lines. The band at the C-line is due to the affinity capture of the biotin portion of the intact reporter by streptavidin, coupled with the detection of the FAM portion by AuNP-conjugated FAM antibody. Because the reporter concentration is low, excess AuNP-conjugated FAM antibody flow to the T-line, where it is captured by the anti-FAM antibody, generating an additional band (Figure 6A, a).

In the second and third scenarios, when the virus is either present in low or high concentration, the activated CRISPR-Cas12a cleaves the target virus genome and also cleaves the reporter. Due to the low concentration of the reporter, all reporter molecules are cleaved, leading to the separation of biotin from FAM. Consequently, no band is generated at the C-line, while a band appears solely at the T-line due to the capture of the AuNP-conjugated FAM antibody (Figures 6A, b, c). Thus, under low reporter concentration, the presence of the virus (regardless of low or high concentration) results in a single band at the T-line, which can be distinguished from a healthy control that produces bands at both the C- and T-lines.

**FIGURE 7:**
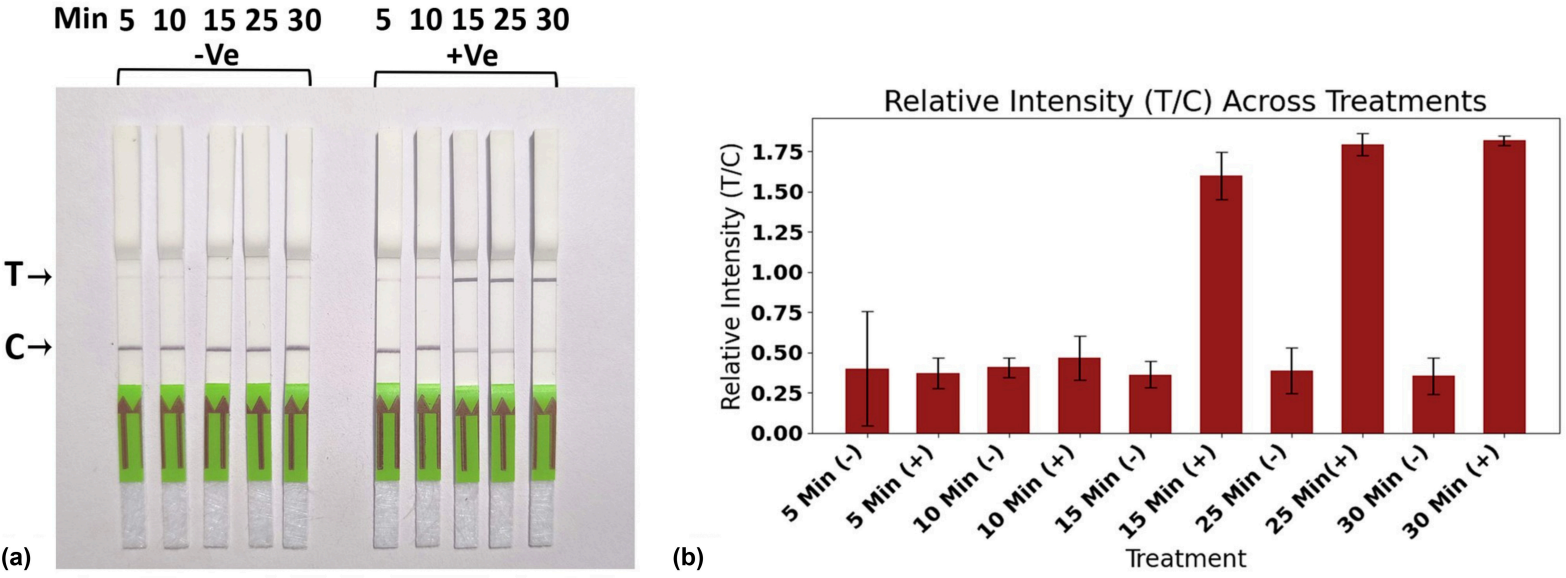
Standardization of reaction time for RPA-CRISPR-cas12a based Lateral flow assay. **(a)** Evaluation of reaction mixture at different reaction times of 5 min, 10 min, 15 min, 25 min, and 30 min. **(b)** Representing relative intensity T/C. All experiments were conducted in triplicates, and representative pictures have been shown. The error bars indicate the standard deviations.

At high reporter concentrations, three similar scenarios can arise, as discussed previously. In the absence of the virus, the CRISPR-Cas12a complex remains inactive, resulting in no cleavage of the reporter. Due to the elevated reporter concentration, all of the AuNP-conjugated FAM antibodies are bound to the FAM portion of the intact reporter, leading to the appearance of a single band in the C-line (Figure 6B, d). In the second scenario, when the virus concentration is low, a smaller number of CRISPR-Cas12a complexes are activated, which cleave a lesser quantity of reporter molecules. Consequently, the intact reporter can still bind to the C-line, while excess AuNP-conjugated FAM antibody is captured at the T-line, producing two bands at the C- and T-lines (Figure 6B, e). Under conditions of higher viral load, there is complete cleavage of all reporter molecules, resulting in a single band appearing at the T-line (Figure 6B, f). Thus, at elevated reporter concentrations, we can distinguish between the absence of the virus, as well as the presence of the virus at both low and high concentrations. Therefore, we chose to continue our experiments with a high reporter concentration of 500 nM. With this reporter concentration, The LFA was further optimized for the incubation time of the reaction, with 25 min identified as the optimal duration (Figures 7a, b).

**FIGURE 8:**
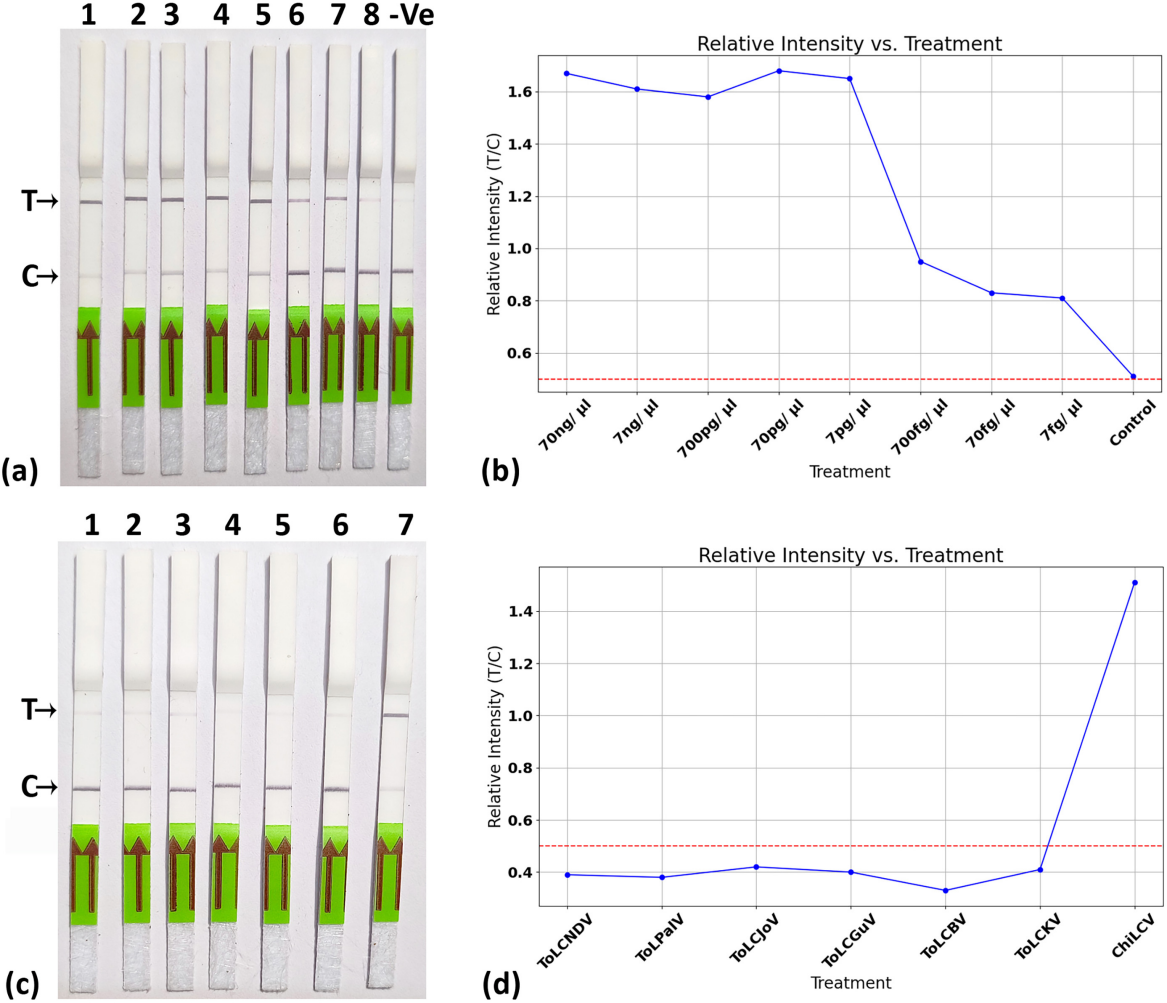
Sensitivity and specificity of CRISPR-Cas12a-based lateral flow system. **(a)** Sensitivity was analyzed using different dilutions of cloned plasmid of ChiLCV at 70 ng/ μl, 7 ng/ μl, 700 pg/ μl, 70 pg/ μl, 7 pg/ μl, 700 fg/ μl, 70 fg/ μl, 7 fg/ μl. The strips were able to detect up to 7 fg/μl. **(b)** Relative intensity measurement for different dilution of target represented with T/C values. **(c)** Specificity assay by testing its efficacy at different virus samples. **(d)** Relative intensity measurement for specificity assay. All experiments were performed in triplicates and representative pictures have been shown.

The detection limit of the LFA was assessed using various dilutions of the cloned viral genome, revealing a threshold detection value of up to 7 fg (Figure 8a). The T/C values for the different dilutions are illustrated in Figure 8b. Additionally, the specificity of the LFA was evaluated using various cloned genomes of closely related begomoviruses, as previously mentioned (Figures 8c, d). The results demonstrated that the LFA system could effectively detect ChiLCV, as indicated by the formation of a band at the T line with the ChiLCV cloned DNA. In contrast, all other cloned begomovirus genomes generated a band at the C-line, with T/C values falling below the threshold value of 0.5.

### 3.5 CRISPR-Cas12a-based LFA assay validated across multi-location field samples, including both symptomatic and asymptomatic plants

**FIGURE 9:**
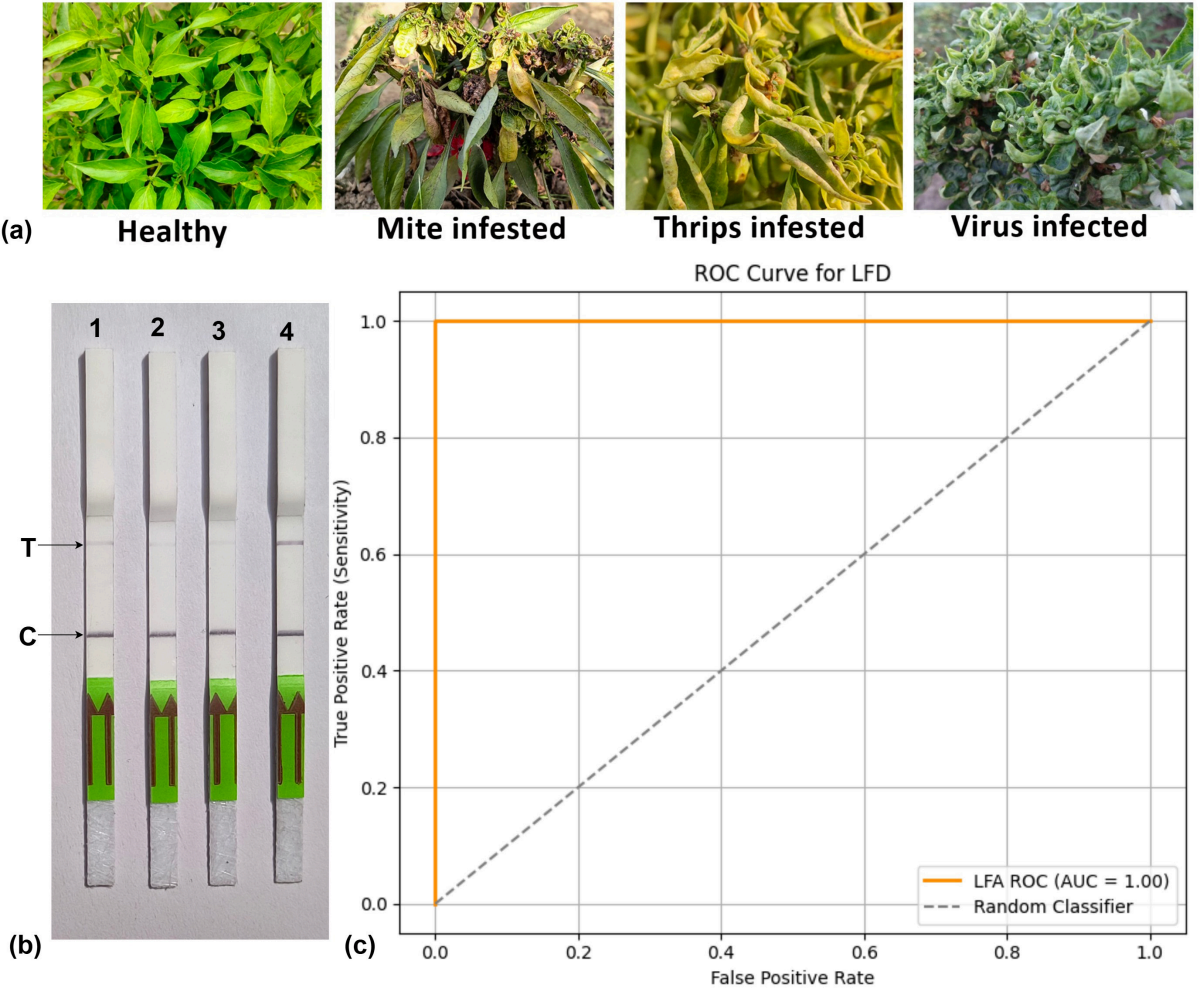
**(a)** Distinguishing symptoms of mite, thrips, and chili leaf curl virus (ChiLCV) infection on chili plants. **(b)** Detection of ChiLCV using CRISPR-cas12a-based-lateral flow strips, Strip 1, 2 and 3: healthy, mite-infested and thrips-infested samples, respectively. **(c)** Representing ROC curve to verify specificity and sensitivity of the system. T/C values calculated in triplicates and representative pictures have been shown.

To assess the practical application of the RPA-Cas12a-based system, comprehensive experiments were conducted using field samples from chili plants infected with mites, thrips, and ChiLCV, all of which exhibit various curling symptoms (Figure 9a). This system successfully detected the ChiLCV virus in the LFA (Figure 9b), ROC = 1 showing high specificity and sensitivity (Figure 9c).

**FIGURE 10:**
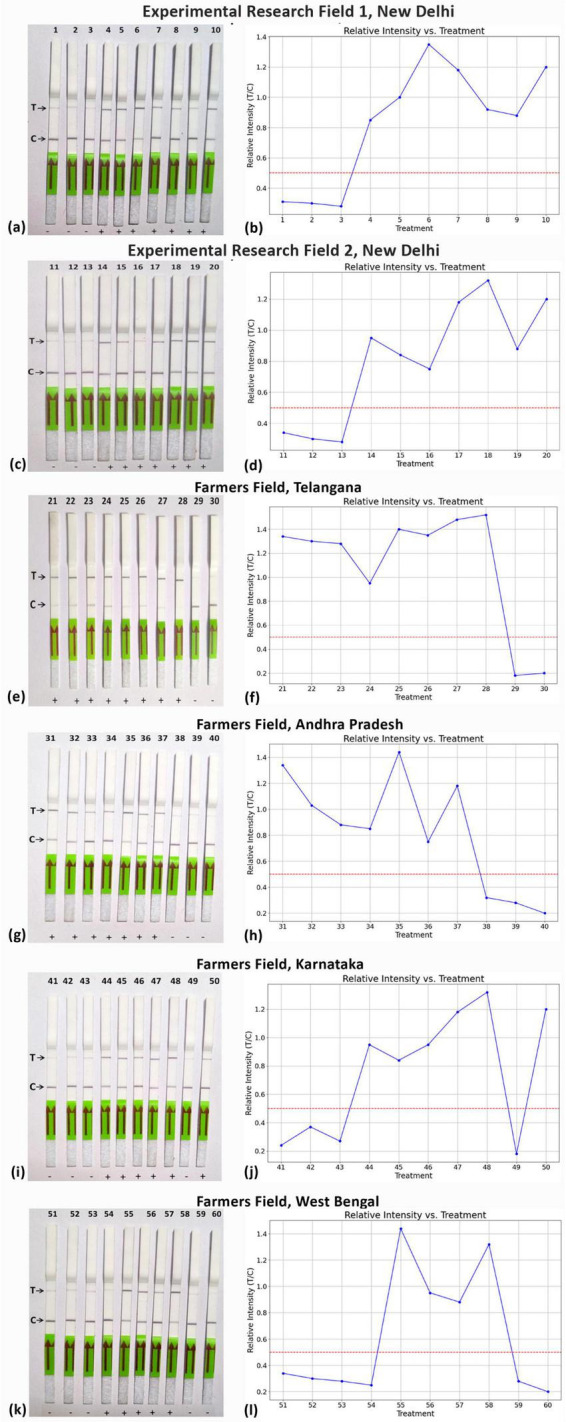
Field-level validation of RPA-assisted CRISPR-Cas12a assay using lateral flow assay from different ChiLCV infected areas. **(a,c,e,g,i,k)** shows lateral flow strip assay conducted from samples collected from research experimental field-1, research experimental field-2, farmers’ field of Telangana, Andra Pradesh, Karnataka and West Bengal, respectively. **(b,d,f,h,j,l)** shows the graphical presentation of relative intensity (T/C) at respective locations.

Moreover, the LFA system was employed to identify ChiLCV in crude leaf extracts from chili plants collected from six distinct locations (Figure 10), including healthy plants as controls. Remarkably, the LFA was capable of detecting ChiLCV in seemingly asymptomatic samples as well as in numerous samples suspected to be infected with either mites or thrips (Table 2). To evaluate the consistency of the diagnostic system across different geographical locations, a chi-square test was performed that compared the number of positive and negative detections at each sampled field location. The analysis revealed no statistically significant difference among locations (χ^2^ = 4.18, df = 5, *p* = 0.5244), suggesting that the diagnostic system exhibits consistent applicability across different regions.

**TABLE 2:** Field level validation of diagnostics developed under the study.

Sate	Location co-ordinates	Symptomatic		Suspected mites/thrips/mixed		Asymptomatic		Total	
		a	b	a	b	a	b	a	b
Delhi	77.170854, 28.641786,	4	4	2	1	4	2	10	7
Delhi	77.157446, 28.642300,	6	6	1	0	3	1	10	7
Telangana	79.713405, 17.712154,	5	5	0	0	5	3	10	8
Andhra Pradesh	78.916456, 14.276932,	4	4	4	3	2	0	10	7
Karnataka	75.260768, 13.850516,	5	5	3	1	2	0	10	6
West Bengal	89.605376, 26.327335,	1	1	0	0	9	3	10	4

a, Total number of samples tested; b, total number of plants showing a positive result.

### 3.6 DETECTR incurs a modestly higher per-sample cost but offers distinct advantages in field applicability

A detailed cost breakdown of ChiLCV diagnostic platforms is presented in Supplementary Table 1. Conventional PCR and qPCR assays were estimated at $4.2 and $4.8–6.0 per sample, respectively, while the crude sap–based RPA-assisted DETECTR assay was calculated at $6.7 per sample. The relatively higher cost of DETECTR arises mainly from the use of commercially sourced Cas12a enzyme and lateral flow strips. By contrast, costs associated with nucleic acid preparation were substantially lower in DETECTR ($0.25) than in PCR/qPCR ($1.80), since the assay bypasses DNA extraction. Equipment amortization was also minimal for DETECTR ($0.01) compared with PCR/qPCR ($0.16–0.40), as only a simple heat block was required.

Despite its slightly higher consumable cost, the DETECTR assay provides several operational advantages: rapid turnaround time (<1 h), elimination of nucleic acid purification, and minimal instrumentation, all of which make it particularly suitable for field deployment. Importantly, with in-house Cas12a production and local fabrication of lateral flow strips, the per-sample cost could be reduced substantially, potentially reaching or even falling below that of qPCR. Thus, DETECTR offers a practical trade-off - slightly higher consumable cost balanced by significantly greater field applicability and scalability in plant virus surveillance.

## 4 Discussion

The study aimed to develop a reliable on-site rapid, highly sensitive, and specific diagnostic for one of the most important begomoviruses, the ChiLCV, which causes several leaf curl disease epidemics in chili growing areas in India. The on-site diagnostic landscape of many begomovirus-induced diseases often relied on simple visual observation of the symptoms. In the case of chili leaf curl disease, the situation is more complex under field conditions, as mites and thrips also account for similar symptom phenotypes, which are often difficult to distinguish at the field level. Due to high sequence conservation at the coat protein gene, ELISA-based diagnostic methods are not suitable for the majority of the begomoviruses. The routine diagnostic methods for ChiLCV detection, including conventional PCR and the gold-standard real-time PCR, are limited in two key aspects: (i) their inability to reliably differentiate among closely related begomoviruses due to the high sequence similarity of their genomes (Kumar et al., 2022), and (ii) their requirement for purified nucleic acid templates, as they cannot directly detect the virus from crude leaf extracts. Owing such inherent complexity of begomovirus detection, diagnostic methodologies often fail to distinguish between closely related begomoviruses (Kumar et al., 2015). In contrast, our CRISPR-based DETECTR assay represents, to the best of our knowledge, the first demonstration of specific and sensitive detection of ChiLCV directly from crude leaf extracts. This feature provides a practical advantage over existing PCR-based approaches, particularly in terms of specificity, sensitivity, and applicability in field conditions. In this study, we separately examine two recent diagnostic methods, namely, RPA and CRISPR-Cas12a for their effectiveness in detection of ChiLCV. Our results showed that the crRNA-Cas12a can specifically detect the ChiLCV as evidenced from *in vitro* cleavage assay and fluorescence-based detection assays (Wang et al., 2023). However, crRNA-Cas12a alone failed to detect the virus from total DNA and from crude leaf extract obtained from infected plants, indicating its limitation on sensitivity. On the other hand, RPA methodology is highly sensitive but lacks specificity for begomovirus detection. Our approach fundamentally addresses the limitations of these two technologies by combining the high-sensitivity amplification of RPA with the unprecedented molecular precision of CRISPR/Cas12a technology. This synergistic methodology not only enhances detection capabilities but also introduces a novel diagnostic paradigm for plant viral detection even from those with no apparent symptoms. The method exhibits high specificity, enabling discrimination of ChiLCV from closely related begomoviruses, which is a challenge for conventional PCR-based diagnostics, The entire process, from crude sap extraction to LFA-based detection, takes a maximum 1 h and can be performed on the field, thus making the system a point-of-care (POC) diagnostic method for the detection of ChiLCV, which provided a practical solution for field-level visualization of the diagnostic result (Shin et al., 2021). This field-deployable method allowed for immediate visualization of results, which is crucial for on-site diagnostics and timely disease management (Rubio et al., 2020). The CRISPR-Cas12a-based LFA DETECTR assay was validated using naturally infected chili samples collected from major chili-growing regions across India. These multi-location samples captured the prevailing genetic diversity of ChiLCV present in farmers’ fields. Importantly, both symptomatic and asymptomatic plants were tested, thereby demonstrating the assay’s robustness in detecting ChiLCV under real-world conditions and its applicability beyond laboratory-inoculated material. The method consistently demonstrated clear differentiation between infected and healthy samples, eliminating the risk of false positives that have plagued previous detection technologies. There was a great level of confusion on the use of LFA for CRISPR-based detection, as many researchers showed the absence of a band represents virus presence (Sun et al., 2024), while others reported differential observation of bands at C- and T-line (Shin et al., 2021). We have demonstrated that the generation of bands in LFA depends on the relative concentration of reporter and virus. When the reporter concentration was low, the virus detection was visualized as the absence of a band in the T-line, while at high reporter concentration, the LFA resulted in either two bands at C- and T-lines, if the virus concentration is low, or one band at T-line, if the virus concentration is high.

## 5 Conclusion

In conclusion, our study adapted and validated the DETECTR system in a plant virology context where its use remains underexplored. By synthesizing advanced molecular technologies, we have developed a diagnostic system by combining the RPA and CRISPR-Cas12a technology that offers a rapid, highly sensitive, and specific solution for detecting ChiLCV. The high sensitivity of RPA ensures early detection, while CRISPR/Cas12a provides the necessary specificity to avoid false positives. The practical application of this technology in an LFA format further enhances its usability for field-level diagnostics, making it an invaluable tool for effective disease management in chili pepper cultivation. The broader scientific significance of this research extends beyond ChiLCV detection. Our integrated approach establishes a conceptual framework for developing next-generation plant virus diagnostic technologies, potentially applicable to a wide range of plant species for adaptive diagnostic platforms in agricultural and phytopathological research. While DETECTR and SHERLOCK assays have indeed been reported for human, animal, and some plant viruses, most implementations remain laboratory-focused and rely on nucleic acid purification. In contrast, our study demonstrates the utility of the DETECTR assay for the first time in direct detection of ChiLCV from crude leaf extracts, thereby advancing its applicability as a practical point-of-care tool for plant virus diagnostics.

## Data Availability

The original contributions presented in this study are included in this article/Supplementary material, further inquiries can be directed to the corresponding authors.

## Footnotes

1
https://www.fao.org/faostat/en/#data/QCL

2
https://www.ncbi.nlm.nih.gov/

3
http://grna.ctegd.uga.edu/

4
http://rna.tbi.univie.ac.at/cgi-bin/RNAWebSuite/RNAfold.cgi
