# Supplementary material for: RPA-assisted CRISPR-Cas12a-enabled point-of-care diagnostic platform for chili leaf curl virus with fluorescent and colorimetric readouts
Source: Front Microbiol. 2025 Oct 15;16:1644322. doi: 10.3389/fmicb.2025.1644322 (PMC12568686; doi:10.3389/fmicb.2025.1644322)
Supplement: Supplementary file 1 [file Table_1.docx]

Supplementary Material


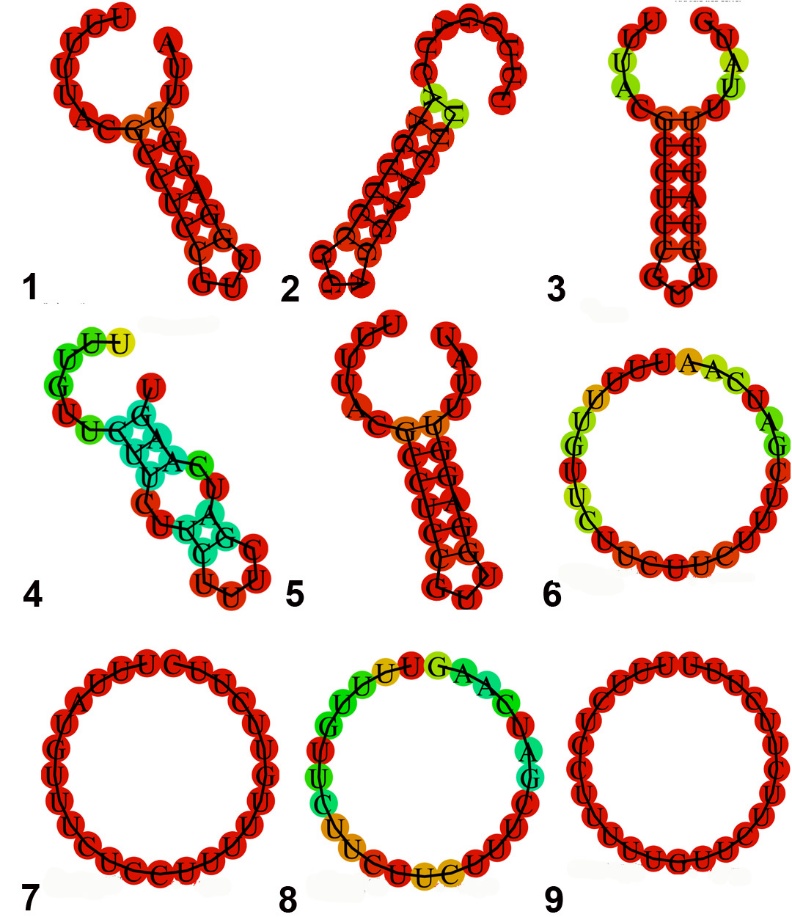


**Figure S1:** Secondary structure of the potential crRNA tested against the target AC1 region of chilli leaf curl virus (ChiLCV). The ranking numbers of the potential crRNA correspond to the ranking represented in Table 1.


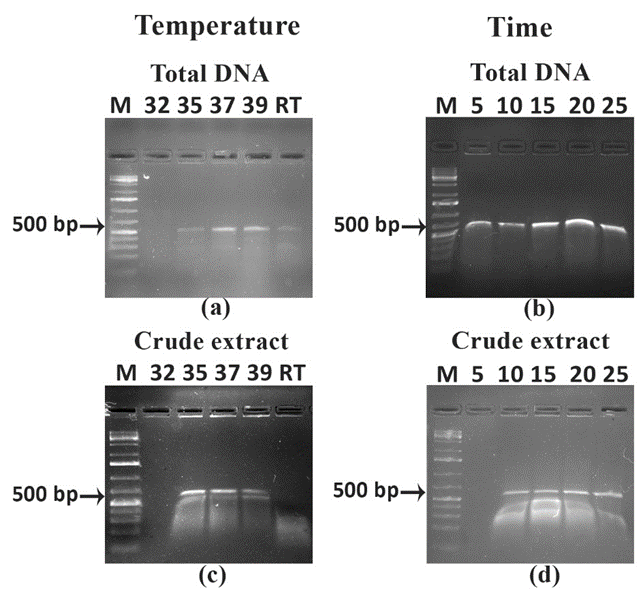


**Figure S2.** Optimization of temperature (a,c) and time (b,d) for RPA assay using total DNA and crude extract obtained from ChiLCV-infected chilli plants.

**Table S1.** Estimated cost per sample (USD) for ChiLCV diagnostics across platforms

| **Cost component** | **Conventional PCR (end-point)** | **qPCR (SYBR Green)** | **qPCR (Probe)** | **DETECTR (crude sap + RPA + LF)** |
| --- | --- | --- | --- | --- |
| Nucleic acid prep | 1.80 | 1.80 | 1.80 | 0.25 (crude buffer) |
| Amplification mix | 0.72 | 1.20 | 2.15 | 3.60 |
| CRISPR/Cas components | – | – | – | 3.11 (Cas12 + crRNA + reporter) |
| Detection readout | 0.48 | – | – | 1.43 (LF strip) |
| Primers/probes | 0.12 | 0.36 | 0.36 | included above |
| Plastics (tips/tubes) | 0.30 | 0.30 | 0.30 | 0.30 |
| Labor (batched, ~24 samples) | 0.15 | 0.15 | 0.15 | 0.09 |
| Overheads (15% consumables) | 0.51 | 0.61 | 0.80 | 1.34 |
| Equipment amortization | 0.16 (PCR + gel doc) | 0.40 (qPCR machine) | 0.40 (qPCR machine) | 0.01 (heat block only) |
| **Total cost per sample** | **4.24** | **4.82** | **5.96** | **6.69** |
